# Antifungal and Antibiofilm Activity of Riparin III against Dermatophytes

**DOI:** 10.3390/jof9020231

**Published:** 2023-02-09

**Authors:** Emanuel Pereira Silva, Marcelo Antônio Nóbrega da Rocha, Risley Nikael Medeiros Silva, Juliana Moura-Mendes, Gabriela Ribeiro de Sousa, Jailton de Souza-Ferrari, José Maria Barbosa-Filho, Edeltrudes de Oliveira Lima, Fillipe de Oliveira Pereira

**Affiliations:** 1 Fungi Research Group, Biochemistry Laboratory, Academic Unit of Health, Education and Health Center, Federal University of Campina Grande, Cuité 58175-000, PB, Brazil; 2 Centro Multidisciplinario de Investigaciones Tecnológicas, Universidad Nacional de Asunción, San Lorenzo 111421, Paraguay; 3 Postgraduate Program in Natural and Synthetic Bioactive Products, Pharmaceutical Sciences Department, Federal University of Paraiba, João Pessoa 58051-900, PB, Brazil; 4 Department of Chemistry, Federal University of Paraiba, João Pessoa 58051-900, PB, Brazil; 5 Department of Pharmaceutical Sciences, Federal University of Paraiba, João Pessoa 58051-900, PB, Brazil

**Keywords:** riparin, dermatophytes, ex vivo, sulfites, antifungal, fungicide, alkamides

## Abstract

The ability of dermatophytes to develop biofilms is possibly involved in therapeutic failure because biofilms impair drug effectiveness in the infected tissues. Research to find new drugs with antibiofilm activity against dermatophytes is crucial. In this way, riparins, a class of alkaloids that contain an amide group, are promising antifungal compounds. In this study, we evaluated the antifungal and antibiofilm activity of riparin III (RIP3) against *Trichophyton rubrum, Microsporum canis*, and *Nannizzia gypsea* strains. We used ciclopirox (CPX) as a positive control. The effects of RIP3 on fungal growth were evaluated by the microdilution technique. The quantification of the biofilm biomass in vitro was assessed by crystal violet, and the biofilm viability was assessed by quantifying the CFU number. The ex vivo model was performed on human nail fragments, which were evaluated by visualization under light microscopy and by quantifying the CFU number (viability). Finally, we evaluated whether RIP3 inhibits sulfite production in *T. rubrum*. RIP3 inhibited the growth of *T. rubrum* and *M. canis* from 128 mg/L and *N. gypsea* from 256 mg/L. The results showed that RIP3 is a fungicide. Regarding antibiofilm activity, RIP3 inhibited biofilm formation and viability in vitro and ex vivo. Moreover, RIP3 inhibited the secretion of sulfite significantly and was more potent than CPX. In conclusion, the results indicate that RIP3 is a promising antifungal agent against biofilms of dermatophytes and might inhibit sulfite secretion, one relevant virulence factor.

## 1. Introduction

Dermatophytoses are mycoses caused by fungi called dermatophytes, divided into seven main clades with the genera *Arthroderma, Lophophyton, Microsporum, Paraphyton, Nannizzia, Epidermophyton,* and *Trichophyton* [1]. Dermatophytes infect humans of all ages, races, genders, and socioeconomic statuses worldwide. *Tinea pedis* and *tinea corporis* have become the principal clinical forms worldwide. *Trichophyton rubrum* predominates globally in cases of *tinea pedis* and *tinea unguium* [2]. Specifically in Brazil, *tinea unguium* (toenails and fingernails) is the most frequent dermatophytosis. *Trichophyton rubrum, M. canis*, and *N. gypsea* have become the major species [3]. Although dermatophytosis is generally restricted to the surface of the skin tissue, fungi can be invasive and cause a deep and widespread infection in patients with autosomal recessive CARD9 deficiency or HIV immunodeficiency or even in patients with unknown immunodeficiency [4,5]. 

The treatment of dermatophytosis aims to reach mycological and clinical cures. The available therapeutic options for treating dermatophytosis comprise a variety of topical and oral antifungals. Oral terbinafine and itraconazole are the treatment of choice. They usually have shorter treatment times and better cure rates but have more significant risks and require closer monitoring [6]. Some topical antifungal options have been developed in the last few years with different posology, associations with physical strategies, and variations in the pharmaceutical formulations [7]. Efinaconazole, tavaborole, and ciclopirox (CPX) are the main efficacious, topical drugs. Therefore, we chose CPX as the standard antifungal in this study. The antifungal mechanism of CPX involves the disruption of fungal metabolism by inhibiting iron-dependent enzymes [8]. 

Even with these treatment options, antifungal resistance has been increasing and causing therapeutic failures. Recently, *T. indotineae* strains with high terbinafine resistance have spread worldwide. They decrease the effectiveness of oral therapy with this antifungal and cause recalcitrant dermatophytosis [9]. The ability of dermatophytes to overcome the host’s immunological mechanisms and histological structure is essential for establishing and worsening the severity of the infection. Dermatophytes produce virulence factors, such as fungal adhesins, fibrils, and lytic enzymes [10], and promote appropriate metabolic adaptations [11] and produce biofilms [12]. Fungal biofilms alter susceptibility to antifungals, contributing to chronicity, recurrence, resistance, and tolerance to conventional drugs. Furthermore, fungal cells in biofilms produce an extracellular matrix, which acts as a physical barrier and drug sink, reducing the effective drug concentration for cells within the biofilm [13].

**Figure 1:**
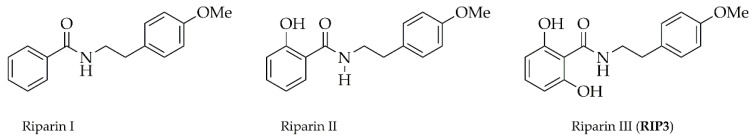
Chemical structures of riparins I, II, and III (RIP3).

Considering this context, research that seeks new drugs for treating dermatophytosis must consider the action against biofilms. In addition, new antifungal drugs that act on virulence factors may contribute to the control of the infection. Riparins are phytochemicals used as model compounds for designing new drugs. They were initially isolated from the fruits of *Aniba riparia* (Nees) Mez (Lauraceae) (Figure 1) [14]. Riparins I, II, and III (RIP3) are alkamide-type alkaloids structurally related to benzamides prepared by chemical synthesis [15]. 

There is a tendency to explore the full range of phytochemicals’ biological potential. In this sense, pharmacological investigations of natural products with multiple biological activities of medicinal interest have increased the importance of developing multi-targeted drugs [16]. For example, several pharmacological activities have been described for RIP3, such as potential antianxiety [17,18], antidepressant [19], and antibacterial activity [20,21]. However, to the best of our knowledge, there has been no report of biological studies on RIP3 against dermatophytes. For this reason, we focused on the antifungal and potential antibiofilm activity of RIP3 against clinically relevant dermatophytes *T. rubrum*, *M. canis*, and *N. gypsea.*

## 2. Materials and Method

### 2.1. Chemistry

All reagents and solvents were purchased from Sigma-Aldrich and were used without further purification. Silica gel matrix, with fluorescent indicator 254 nm, was used in analytical thin-layer chromatography. The reaction and purification were monitored by TLC. All evaporations were performed under reduced pressure. Yields refer to chromatographically and spectroscopically (1H and 13C NMR) homogeneous material. IR spectra are reported in wavenumbers (cm-1) and were recorded on an IR Prestige-21 FTIR (Shimadzu, Kyoto, Japan) spectrophotometer using attenuated total reflectance (ATR). High-resolution mass spectrometry (HRMS) analysis was performed by direct infusion in the mass spectrometer using electrospray ionization with a quadrupole time-of-flight analyzer on a microTOF-QII ESI-TOF (Bruker Daltonics, Billerica, MA, USA) mass spectrometer. Nuclear magnetic resonance (NMR) experiments were performed in a Bruker Avance III HD instrument (400 MHz for 1H and 100 MHz for 13C) using CDCl_3_ as solvent and TMS as the internal standard. Chemical shifts are expressed as d values in parts per million (ppm) from TMS (0 ppm) and coupling constant (J) in hertz (Hz). Abbreviations to denote the multiplicity of a particular signal are s (singlet), d (doublet), t (triplet), q (quartet), m (multiplet), and sl (signal large).

### 2.2. Synthesis

In a 50 mL round-bottom flask equipped with a magnetic stir bar containing a solution of methyl 2,6-dihydroxybenzoate (0.60 mmol; 1 equiv) in DCM (10 mL), we added 4-methoxyphenethylamine (1.22 mmol; 2 equiv). After, the resulting reaction mixture was stirred for 4 h at room temperature. After completion, the solvent was removed under reduced pressure. We dissolved the crude material in ethyl acetate and washed it successively with an aqueous 1% HCl solution (3 × 10.0 mL) and water (2 × 10.0 mL). The organic layer was dried with Na_2_SO_4_, filtered, and concentrated under reduced pressure. The resulting crude product was purified by flash column chromatography on silica gel and eluted with ethyl acetate and hexane mixtures, providing pure riparin III (RIP3; 2,6-dihydroxy-N-(4-methoxyphenethyl)benzamide) as a pearly white solid (30% yield) spectroscopically identical to that previously reported [14,15].

2,6-dihydroxy-N-(4-methoxyphenethyl)benzamide (RIP3): melting point 107–108 °C; IR (ATR) ν/cm^−1^ 3340, 3070, 1581; ^1^H RMN (400 MHz, CDCl_3_) ẟ 10,58 (sl, 2H), 8.47 (sl, 1H), 7.14–7.08 (m, 3H), 6.82 (d, *J* 8.0 Hz, 2H), 6.37 (d, *J* 8.0 Hz, 2H), 3.76 (s, 3H), 3.64 (q, *J* 12.0 Hz, 2H), 2.82 (t, *J* 12.0 Hz, 2H); ^13^C RMN (101 MHz, CDCl_3_) ẟ 170.15, 158.24, 133.19, 130.90, 129.76, 114,05, 108.26, 103.17, 55.27, 40.90, 34.50; HRMS *m/z*, calc. for C_16_H_16_NO_4_ [M − H]^−^: 286.1090, found: 286.1085. 

### 2.3. Drugs Dilutions

RIP3 and ciclopirox (CPX) (Sigma-Aldrich^®^, St. Louis, MO, USA) were dissolved in dimethylsulfoxide (DMSO) and diluted in RPMI 1640 medium (Sigma-Aldrich^®^) to reach 1024 μg/mL. We used ciclopirox (Sigma-Aldrich^®^) as a conventional antifungal. The highest concentration of DMSO used was 0.5%. We performed serial dilutions in RPMI 1640 medium to reach lower concentrations.

### 2.4. Antifungal Susceptibility Testing

#### 2.4.1. Fungi

We used the following fungal strains for the antifungal study (Table 1), obtained from the collection of the Mycology Laboratory of the Department of Pharmaceutical Sciences, Federal University of Paraíba (Brazil): *Trichophyton rubrum* ATCC 28188; *T. rubrum* LM 03 (scalp); *T. rubrum* LM 06 (nails); *T. rubrum* LM 63 (nails); *T. rubrum* LM 70 (scalp); *T. rubrum* LM 176 (thorax); *Microsporum canis* ATCC 36295; *M. canis* LM 177 (scalp); *M. canis* LM 186 (arms); *M. canis* LM 216 (scalp); *M. canis* LM 232 (feet); *M. canis* LM 665 (head); *Nannizzia gypsea* ATCC-24102; *N. gypsea* LM-5 (toenail); *N. gypsea* LM-129 (legs); *N. gypsea* LM-130 (legs); *N. gypsea* LM-184 (feet); and *N. gypsea* LM-305 (feet). The fungal strains were cultured on potato glucose agar (Difco^®^, Franklin Lakes, NJ, USA) at 28 °C for up to 7 days. Recent fungal colonies were covered with sterile saline (0.9% NaCl) and shaken lightly. The inocula densities were adjusted in a spectrophotometer at 530 nm for a 70–72% transmittance value. Subsequently, each inoculum was diluted in RPMI 1640 medium to obtain a final concentration of 0.4 x 10^3^ to 5 × 10^3^ CFU/mL [22].

#### 2.4.2. Minimum Inhibitory Concentration (MIC)

We determined the MIC values of RIP3 and CPX by the microdilution technique [22]. In each plate row, we added 100 µL of the test drugs diluted in RPMI 1640 (pH 7.0). Afterwards, we added 100 µL of the inoculum to each well. A negative control (RPMI 1640 + inoculum) and a control with DMSO (DMSO + inoculum + RPMI 1640) were performed. The plates were sealed and incubated at 28 °C for up to 7 days for reading. MIC is defined as the lowest concentration of drugs capable of visually inhibiting fungal growth compared to the control [22].

#### 2.4.3. Minimum Fungicide Concentration (MFC)

Aliquots of 10 μL were taken from each well at concentrations higher than the MIC and plated on Sabouraud glucose agar plates (Difco^®^). Fungal colonies were counted after incubating the plates at 28 °C for up to 7 days. MFC is the lowest drug concentration that resulted in fewer than three colony growths (99.9% death) [23]. A drug demonstrates fungicidal action when the MFC/MIC ratio does not exceed the value of 4, while it is considered fungistatic when MFC/MIC is greater than 4 [24].

### 2.5. Biofilm Assays

#### 2.5.1. In Vitro Biofilm Formation

We performed the in vitro biofilm formation assay on 96-well polystyrene plates, as described by Brilhante et al., 2018 [25]. An aliquot of 100 μL of the fungal inoculum (1 × 10^6^ CFU/mL) was added to the wells of the plate and incubated at 37 °C for 3 h for biofilm pre-adhesion. After washing with sterile saline to remove non-adherent cells, 200 μL RPMI 1640 was added. We incubated the plates at 37 °C without shaking for 72 h for biofilm formation. After incubation, the culture medium was removed from each well, and the plates were rewashed. After drying the plates, 100 μL of crystal violet solution (ethanol 0.5%) was added to each well and incubated for 20 min. The plates were washed, and the biofilms were decolorized with 99.8% ethanol. Finally, we read the wells at 570 nm [26]. Based on these results, we classified each strain into different degrees of biofilm production. We defined the optical density (OD) cut-off value concerning the negative control values (RPMI 1640, without biofilm). The cut-off value (ODc) was defined as three standard deviations above the mean OD 570 nm of the negative control. Strains were classified as non-producing (OD ≤ ODc), weak (ODc < OD ≤ 2 × ODc), moderate (2 × ODc < OD ≤ 4 × ODc), and strong biofilm producers (4 × ODc < OD) [27].

#### 2.5.2. In Vitro Biofilm Inhibition

We performed the previous test in the presence of RIP3 and CPX. After the pre-adhesion period, 200 μL RPMI 1640 with the test drugs (MIC, 2xMIC, 4xMIC, and 8xMIC) was dispensed into the wells. We performed the control with RPMI 1640. The plates were incubated at 37 °C without shaking for 72 h. Biofilm biomass quantification was evaluated by 0.5% crystal [28]. After biofilm formation (72 h), we added 100 μL of sterile saline to each well, followed by vigorous shaking to suspend the cells from the biofilm entirely. The suspensions were diluted 1:10 in sterile saline, and a 10 µL aliquot was put onto the surface of a plate containing Sabouraud glucose agar. The plates were incubated at 28 °C for seven days for CFU counting [29].

#### 2.5.3. Ex Vivo Biofilm Inhibition

The nail fragments collected were disinfected with ethanol for 15 min, dried at 28 °C, and sterilized in an autoclave (1 atm, 120 °C, 15 min). The sterilized material was kept in sealed tubes until used in the experiments. Nail fragments (2 mm) were dispensed into wells of 96-well plates and covered with 50 μL of the fungal inoculum at 28 °C for three hours. Then, 50 μL of the test drugs (MIC, 2xMIC, 4xMIC, and 8xMIC) was added. We performed two controls: (1) without drugs and (2) without inoculum and drugs. The plates were incubated at 28 °C for 7 days. The biofilm was analyzed by optical microscopy to visualize the biofilm on the nail fragments. We used light microscopy to characterize fungal structures grown on nail fragments, such as individual conidia associated with entangled fungal hyphae. We analyzed 10 microscopy fields per slide and recorded the structures as strong (+++), intermediate (++), low (+), or absent (−) [30]. In addition, ex vivo biofilms were subjected to CFU counting. The suspensions were diluted 1:10 in sterile saline, and 10 µL was seeded onto the surface of a plate containing Sabouraud glucose agar. The plates were incubated at 28 °C for seven days for CFU counting [29]. We did not detect differences in growth rates between nails from different individuals.

### 2.6. Sulfite Detection

Initially, we performed an assay to verify the sensitivity of dermatophytes to cysteine. An aliquot of 50 µL of the inocula was placed onto the surface of agar plates with M9 medium (Sigma-Aldrich^®^) supplemented with 0.5% glucose, 1.5% agar, and 50 mM L-cysteine. The plates were incubated for seven days at 28 °C to verify the presence or absence of growth under these conditions. The next step of sulfite detection was performed with the strains that grew in M9 with L-cysteine. For this, tubes containing 0.5 mL of the fungal inoculum were incubated with 9.5 mL of M9 medium (broth) supplemented with 10 mM L-cysteine. The material was incubated for seven days at 28 °C and centrifuged at 1000× *g* for 3 min. According to the manufacturer’s instructions, the supernatant was used for the sulfite detection kit (Sigma-Aldrich^®^). This assay is based on sulfite oxidation to sulfate producing a stable signal at 570 nm [31]. 

### 2.7. Statistical Analysis

The experiments were performed in three independent experiments. MIC and MFC values were expressed as mode, and the results of biofilm assays were expressed as mean ± SD (standard deviation). We performed one-way ANOVA to determine significant differences (*p* < 0.05) between treatments with Tukey’s test. The confidence interval was 95%. Data treatment was performed in R 4.1.0 in the RStudio interface, and we used the ggplot2 package for data visualization.

## 3. Results

MIC and CFM values of CPX and RIP3 are shown in Table 1. RIP3 inhibited the growth of *T. rubrum* and *M. canis* at 128 mg/L and *N. gypsea* at 256 mg/L. It presented fungicide action. Ciclopirox showed fungicide action against 99% of the tested strains. We chose *T. rubrum* ATCC 28188, *M. canis* ATCC 36295, and *N. gypsea* ATCC 24102 strains for the following assays to evaluate the effects of RIP3 on biofilm in vitro and ex vivo models. These strains were sensitive to RIP3 and strong/moderate biofilm producers on in vitro biofilm models (Table 1).

**Figure 2:**
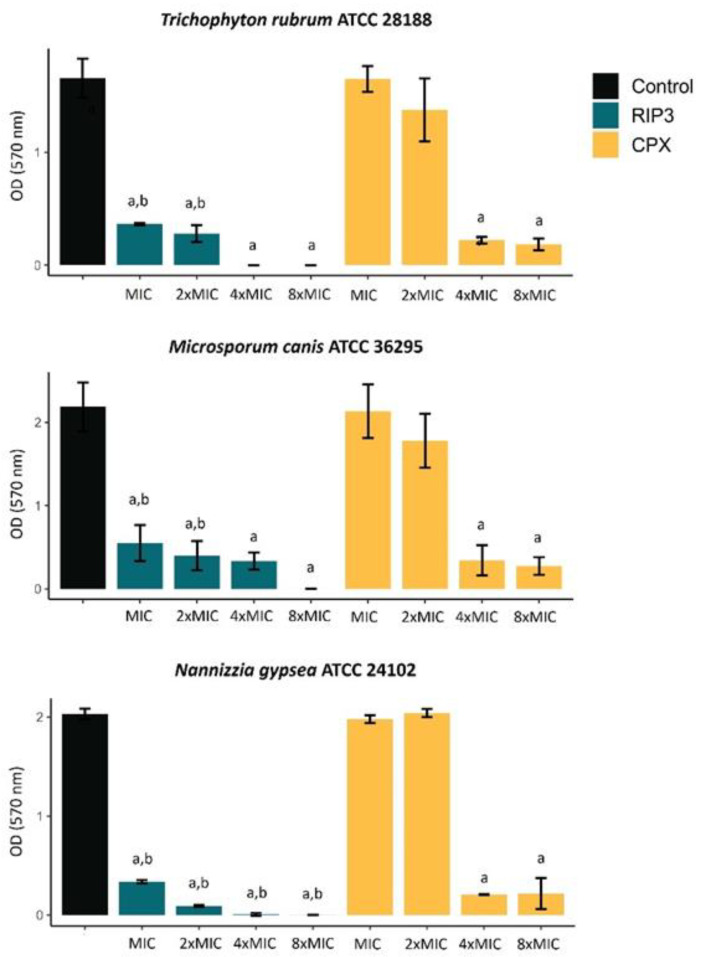
Effect of RIP3 on the biofilm biomass of dermatophytes expressed as absorbance of the crystal violet staining. The results are the means ± SD from three independent experiments. Significant difference (*p* < 0.05) when compared to drug-free growth control (a) and to CPX at the respective MIC value (b). OD, optical density; MIC, minimal inhibitory concentration; RIP3, riparin III; CPX, ciclopirox.

The quantification of in vitro biofilm mass in the presence and absence of drugs was expressed in optical density (OD) values at 570 nm (Figure 2). RIP3 significantly inhibited the biofilm formation by *T. rubrum, M. canis*, and *N. gypsea* at MIC (*p* < 0.05). Although CPX had much lower MIC values than RIP3 (Table 1), CPX showed effectiveness only at 4xMIC (*p* < 0.05). The mean percentage of inhibition ranged from 69 to 99% compared to the control.

**Figure 3:**
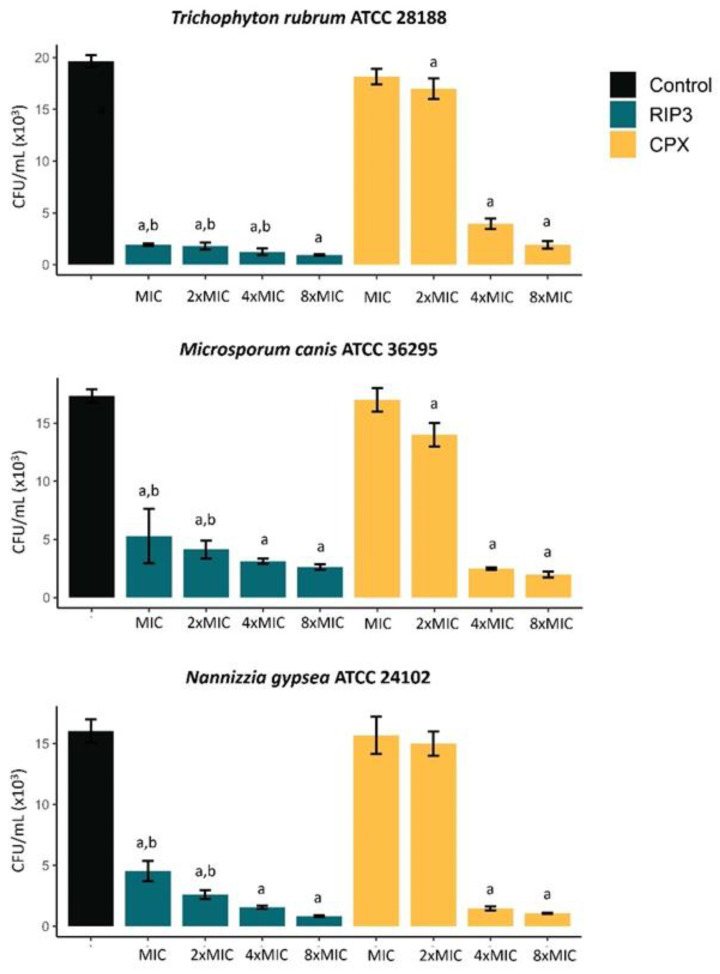
Effect of RIP3 on the viability of in vitro biofilms of dermatophytes determined by quantifying the number of colony-forming units (CFU). The results are the means ± SD from three independent experiments. Significant difference (*p* < 0.05) when compared to drug-free growth control (a) and to CPX at the respective MIC value (b). OD, optical density; MIC, minimal inhibitory concentration; RIP3, riparin III; CPX, ciclopirox.

We analyzed biofilm viability in vitro by CFU counting; the data are shown in Figure 3. Following the pattern of the results found in the previous test, RIP3 significantly reduced biofilm viability at the MIC (*p* < 0.05). CPX reduced biofilm viability, starting with 4xMIC (*p* < 0.05). The mean percentage of viability reduction ranged from 69 to 95% compared to the control.

**Figure 4:**
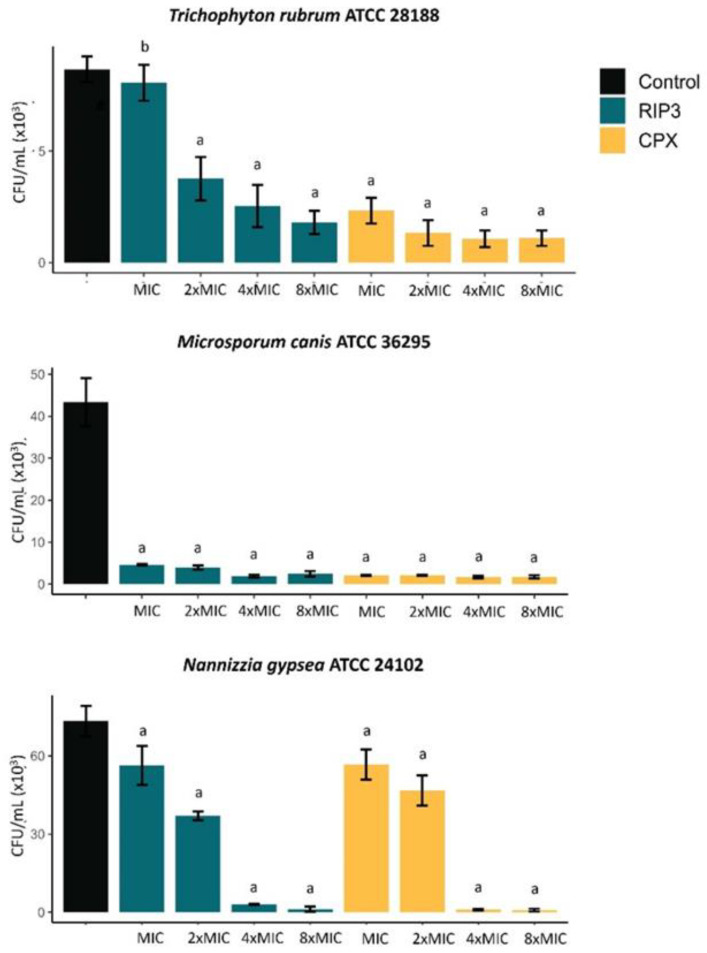
Effect of RIP3 on the viability of ex vivo biofilms (nail fragments) of dermatophytes determined by quantifying the number of colony-forming units (CFU). The results are the means ± SD from three independent experiments. Significant difference (*p* < 0.05) when compared to drug-free growth control (a) and CPX at the respective MIC value (b). OD, optical density; MIC, minimal inhibitory concentration; RIP3, riparin III; CPX, ciclopirox.

We evaluated the antibiofilm potential of RIP3 using ex vivo models by visualizing the structures under light microscopy (Table 2) and quantifying the number of CFU (Figure 4). RIP3 and CPX at 4xMIC and 8xMIC visually inhibited the fungal growth on nail fragments (Table 2). RIP3 significantly reduced ex vivo biofilm viability of *M. canis* and *N. gypsea* at the MIC (*p* < 0.05) (Figure 4). The biofilm viability of *T. rubrum* was inhibited at 2xMIC. The average percentage of viability reduction ranged from 50 to 98% compared to the control. However, RIP3 was less effective than CPX at its respective MIC value (*p* < 0.05).

## 4. Discussion

Research into new antifungals may provide the most promising advances in the coming years for treating dermatophytosis. In this way, our results demonstrated that RIP3 showed antifungal activity against dermatophyte strains as a fungicide. Furthermore, RIP3 also exerted a relevant inhibitory action on dermatophyte biofilms using in vitro (polystyrene plates) and ex vivo (nail fragments) experimental models. Finally, we used a strain of *T. rubrum* as a model to study the action of RIP3 on its sulfite secretion.

In the literature, we found no studies describing the antifungal and antibiofilm activity of RIP3 against dermatophytes. However, we found that RIP3 showed better antibacterial activity against Gram-positive than Gram-negative bacteria [20]. In a previous study, *S. aureus* strains were more sensitive to RIP3 than *Escherichia coli* strains [21]. RIP3 exhibited antimicrobial activity in multidrug-resistant *S. aureus* and *Acinetobacter baumannii* and promoted ultrastructural change. In addition, RIP3 showed potential antibiofilm produced by *S. aureus* [20].

RIP3 and CPX showed inhibitory effects against dermatophyte strains. However, effective concentrations differed between drugs, and MIC values of RIP3 were higher than those of CPX. Most MIC values of other antifungals used to treat dermatophytosis are usually low. Specifically, studies revealed that the MIC of CPX ranges from 0.25 to 8 mg/L [32,33]. High inhibitory concentrations can lead to greater use of drugs in pharmaceutic formulations and undesirable effects. Due to this observation, we searched for studies that analyzed the toxicity of RIP3. RIP3 induced cytotoxicity and hemolysis similar to oxacillin and meropenem, two antimicrobials used in clinical practice [20]. In a study performed with embryos of *Gallus gallus*, morphological alterations were observed in all the tested concentrations of RIP3. However, there were no changes in the structures’ functions, which were considered potentially safe for pregnant women [34]. RIP3 showed intraperitoneal toxicity (LD50: 104.2 mg/kg). RIP3 (500 mg/kg orally or 35 mg/kg intraperitoneally) did not induce behavioral changes in mice [35].

Although RIP3 might be safe in cellular toxicity assays, the administration of RIP3 warrants caution when administered systemically. The combination of drugs can reduce these effects and accelerate clinical and microbiological improvement [36]. Antifungal combinations in dermatophytes have elicited considerable scientific interest over the years. However, no study has focused on combinations of RIP3 and conventional drugs. Then, it is relevant to continue research using RIP3 as a drug prototype.

Most studies investigating the in vitro antibiofilm activity in dermatophytes were performed with conventional drugs such as terbinafine, griseofulvin, econazole, fluconazole, itraconazole, voriconazole, and ciclopirox [37]. Commonly, the effective concentrations of conventional drugs against dermatophyte biofilms in vitro were higher than the MIC (planktonic cells). However, surprisingly, our study revealed inhibitory effects on biofilm formation and viability by RIP3 at the MIC.

Considering the evidence about the pharmacological activity and toxicity of RIP3, we performed assays using human nail fragments as an ex vivo biofilm model. Ex vivo experimental models are fundamental for evaluating new drugs once they attempt to mimic in vivo conditions [38]. We chose human nail fragments as an ex vivo model based on three points: (1) RIP3 showed toxicity in animal models when administered intraperitoneally [35]; (2) topical use of antifungals may be safer than oral therapy [39]; (3) the potential topical use of formulations with RIP3 makes future studies of combined therapy possible [40]. Our results showed that RIP3 shows potential biofilm inhibition that could be an option for treating topical dermatophytosis.

Biofilms in nail dermatophytosis are related to the resistance to injury and may act as a constant source of infection, contributing to antifungal resistance. Moreover, fungal biofilms influence the permeation of topical treatment [41]. Therefore, designing new antifungals for topical treatment is crucial for targeting biofilms. In a comprehensive review, Gupta et al. (2021) provided an excellent update on antifungal agents and new formulations against dermatophytes [40]. In this study, we saw that topical formulations for treating *tinea unguium*, such as amorolfine 5% and ciclopirox 8% nail lacquers, have been used for a long time. Indeed, topical formulations, such as nail lacquer forms, improve the efficacy of the treatment by staying in contact with the nail plate for long periods, delivering the drug to the infected site and reaching the fungi at an effective concentration [7].

Dermatophyte virulence factors are another target for assessing the activity of new drugs because they help fungi to attack the host tissue. With the antibiofilm potential of RIP3 established, we investigated whether RIP3 interferes with a vital virulence factor involved in the degradation of host keratin: production and release of sulfites. The details can be found in the Supplementary Material (Figure S1). Keratinolytic dermatophyte proteases have a relevant role in the pathogenesis of dermatophytosis. Therefore, dermatophytes need to express secreted proteases to break down keratins in human tissues to exploit this nitrogen source and survive [42].

The keratinolytic process in nail plates depends on sulfitolysis, a process that induces the cleavage of the disulfide bridges (S–S), changing the conformation of keratins and facilitating the action of keratinase. This process can occur through sulfite^-^, compounds that interact with cysteine residues in keratins and promote cross-linking [43]. Previously, Grumbt et al. (2013) proposed that dermatophytes secrete sulfites through the efflux pump Ssu1 during growth in an environment rich in cysteine [31]. The excess of free cysteine may be toxic to the fungi and the host’s tissues. However, fungi reuse metabolized sulfite as a defense mechanism. In this study, we used *T. rubrum* ATCC 28188 in this step because it grew in M9 medium supplemented with L-cysteine (Figure S1).

We considered these assumptions to present two main points concerning the antibiofilm activity of RIP3. First, we confirmed that RIP3 has antibiofilm activity, supported by intense activity in vitro and ex vivo using nail fragments as a source of keratin. Second, RIP3 decreases sulfite formation during fungal growth in an L-cysteine-rich environment. However, broadening the number of dermatophyte strains and virulence factors is relevant for consolidating the RIP3 interference mechanism in biofilm formation.

The mechanisms involved in nail infection and drug permeation must be investigated to find practical solutions for dermatophytosis treatment. Our study attempts to overcome this scenario by presenting a solid candidate drug in combating biofilm development: RIP3. We provide the original hypothesis that the antibiofilm action of RIP3 might inhibit the secretion of the reducing agent sulfite. The present study is the first evidence that reports the potential of RIP3 to control dermatophytes biofilms.

## Figures and Tables

**Table 1:** MIC and MFC values (mg/L) of RIP3 and CPX against dermatophytes strains.

Fungi	RIP3			CPX			Biofilm Production
	MIC	MFC	MFC/MIC	MIC	MFC	MFC/MIC	
*Trichophyton rubrum* ATCC 28188	256	256	1	1	2	2	Strong
*Trichophyton rubrum* LM 03	128	128	1	1	2	2	Strong
*Trichophyton rubrum* LM 06	128	128	1	1	2	2	Moderate
*Trichophyton rubrum* LM 63	512	1024	2	1	2	2	Strong
*Trichophyton rubrum* LM 70	256	256	1	0.5	1	2	Strong
*Trichophyton rubrum* LM 176	256	256	1	0.5	1	2	Weak
*Microsporum canis* ATCC 36295	128	1024	8	1	2	2	Moderate
*Microsporum canis* LM 177	128	512	4	1	2	2	Strong
*Microsporum canis* LM 186	128	512	4	1	2	2	Strong
*Microsporum canis* LM 216	128	128	1	1	2	2	Strong
*Microsporum canis* LM 232	128	256	2	1	2	2	Moderate
*Microsporum canis* LM 665	128	1024	8	2	2	1	Strong
*Nannizzia gypsea* ATCC 24102	256	512	2	2	2	1	Strong
*Nannizzia gypsea* LM 5	256	1024	4	2	2	1	Strong
*Nannizzia gypsea* LM 129	256	512	2	2	2	1	Strong
*Nannizzia gypsea* LM 130	256	1024	1	2	2	1	Moderate
*Nannizzia gypsea* LM 184	256	1024	4	2	32	16	Strong
*Nannizzia gypsea* LM 305	256	256	1	2	2	1	Moderate

MIC, minimal inhibitory concentration; MFC, minimal fungicide concentration; RIP3, riparin III; CPX, ciclopirox.

**Table 2:** Ex vivo production of biofilm (nails fragments) by dermatophytes in the absence (control) and presence of RIP3.

Drugs	*Trichophyton rubrum* ATCC 28188	*Microsporum canis* ATCC 36295	*Nannizzia gypsea* ATCC 24102
Control	+++	+++	+++
RIP3 MIC	+	+	+
RIP3 2xMIC	+	+	+
RIP3 4xMIC	−	−	−
RIP3 8xMIC	−	−	−
CPX MIC	++	++	++
CPX 2xMIC	+	+	+
CPX 4xMIC	−	−	−
CPX 8xMIC	−	−	−

MIC, minimal inhibitory concentration; RIP3, riparin III; CPX, ciclopirox. +++ strong; ++ intermediate; + low; − absent.

## Data Availability

Not applicable.

## Supplementary Materials

The following supporting information can be downloaded at: https://www.mdpi.com/article/10.3390/jof9020231/s1, Figure S1: effect of RIP3 on sulfite formation by *Trichophyton rubrum* ATCC 28188.
