# Supplementary material for: Antifungal and Antibiofilm Activity of Riparin III against Dermatophytes
Source: J Fungi (Basel). 2023 Feb 9;9(2):231. doi: 10.3390/jof9020231 (PMC9966229; doi:10.3390/jof9020231)
Supplement: Supplementary file 1 [file jof-09-00231-s001.zip › jof-2087137-supplementary.pdf]

Supplementary Material

Sulfite detection

Initially, we performed an assay to verify the sensitivity of dermatophytes to cysteine. An aliquot of 50 μL of the inocula was placed onto the surface of agar plates with M9 medium (Sigma-Aldrich®) supplemented with 0.5% glucose, 1.5% agar, and 50 mM L-cysteine. The plates were incubated for seven days at 28°C to verify the presence or absence of growth under these conditions. The next step of sulfite detection was performed with the strains that grew in M9 with L-cysteine. For this, tubes containing 0.5 mL of the fungal inoculum were incubated with 9.5 mL of M9 medium (broth) supplemented with 10 mM L-cysteine. The material was incubated for seven days at 28ºC and centrifuged at 1000g for 3 minutes. According to the manufacturer's instructions, the supernatant was used for the sulfite detection kit (Sigma-Aldrich®). This assay is based on sulfite oxidation to sulfate producing a stable signal at 570 nm [32].

The detection of sulfite assay was performed with *T. rubrum* ATCC 28188 as a fungal model. We excluded *M. canis* ATCC 36295 and *N. gypsea* ATCC 24102 strains on detection of sulfite assay because they did not grow in M9 medium supplemented with L-cysteine. The excess of free L-cysteine may be toxic to the fungi; therefore, analyzing the survival of the strains in media enriched with L-cysteine is a relevant assumption. RIP3 inhibited the formation and secretion of sulfite significantly ($p < 0.05$) and was more potent than CPX (Figure S1).

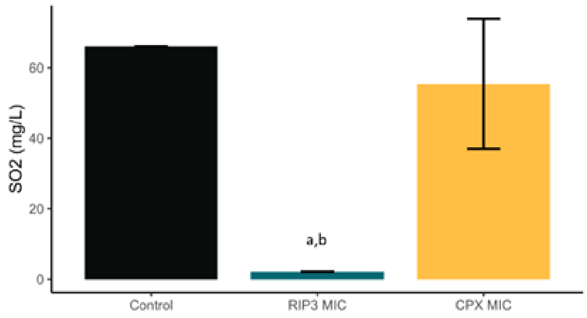

Figure S1. Effect of RIP3 on sulfite formation by *Trichophyton rubrum* ATCC 28188. The results are the means ±SD from three independent experiments. Significant difference ($p < 0.05$) when compared to drug-free growth control (a) and CPX (b). MIC, minimal inhibitory concentration; RIP3, riparin III; CPX, ciclopirox.
